# SARS-CoV-2 and arbovirus infection: a rapid systematic review

**DOI:** 10.1590/1516-3180.2020.0422.08092020

**Published:** 2020-10-20

**Authors:** Keilla Martins Milby, Alvaro Nagib Atallah, César Ramos Rocha-Filho, Ana Carolina Pereira Nunes Pinto, Aline Pereira da Rocha, Felipe Sebastião de Assis Reis, Nelson Carvas, Vinicius Tassoni Civile, Rodolfo Rodrigo Pereira Santos, Laura Jantsch Ferla, Giulia Fernandes Moça Trevisani, Gabriel Sodré Ramalho, Maria Eduarda dos Santos Puga, Virgínia Fernandes Moça Trevisani

**Affiliations:** I PhD. Nurse, Volunteer Researcher at Cochrane Brazil, São Paulo (SP), Brazil.; II MD, MSc, PhD. Nephrologist and Full Professor, Discipline of Emergency and Evidence-Based Medicine, Escola Paulista de Medicina (EPM), Universidade Federal de São Paulo (UNIFESP), São Paulo (SP), Brazil; Director, Cochrane Brazil, São Paulo (SP), Brazil.; III MSc. Biotechnologist and Doctoral Student, Evidence-Based Health Program, Universidade Federal de São Paulo (UNIFESP), São Paulo (SP), Brazil.; IV MSc. Physiotherapist and Doctoral Student, Evidence-Based Health Program, Universidade Federal de São Paulo (UNIFESP), São Paulo (SP), Brazil; Professor, Biological and Health Sciences Department, Universidade Federal do Amapá (UNIFAP), Amapá (AP), Brazil; Volunteer Researcher, Cochrane Brazil, São Paulo (SP), Brazil.; V MSc. Pharmacist and Doctoral Student, Evidence-Based Health Program, Universidade Federal de São Paulo (UNIFESP), São Paulo (SP), Brazil; Volunteer Researcher, Cochrane Brazil, São Paulo (SP), Brazil.; VI MD, MPS. Manager, Medical Practices, Beneficência Portuguesa de São Paulo, São Paulo (SP), Brazil.; VII Physical Educator, Universidade Ibirapuera, São Paulo (SP), Brazil.; VIII MSc. Physiotherapist and Doctoral Student, Evidence-Based Health Program, Universidade Federal de São Paulo (UNIFESP), São Paulo (SP), Brazil; Assistant Professor, Physiotherapy Course, Universidade Paulista (UNIP), São Paulo (SP), Brazil; Volunteer Researcher, Cochrane Brazil, São Paulo (SP), Brazil.; IX Statistician and Master's Student, Evidence-Based Health Program, Universidade Federal de São Paulo (UNIFESP), São Paulo (SP), Brazil; Data Science Manager, Synova Health CRO, São Paulo (SP), Brazil.; X Undergraduate Medical Student, Universidade Federal de São Paulo (UNIFESP), São Paulo (SP), Brazil.; XI Undergraduate Medical Student, Universidade Santo Amaro (UNISA), São Paulo (SP), Brazil.; XII Undergraduate Medical Student, Escola Paulista de Medicina (EPM), Universidade Federal de São Paulo (UNIFESP), São Paulo (SP), Brazil.; XIII MSc, PhD. Librarian, Evidence-Based Health Program, Universidade Federal de São Paulo (UNIFESP), São Paulo (SP), Brazil.; XIV MD, MSc, PhD. Rheumatologist, Discipline of Emergency and Evidence-Based Medicine, Escola Paulista de Medicina (EPM), Universidade Federal de São Paulo (UNIFESP), São Paulo (SP), Brazil; Rheumatologist, Discipline of Rheumatology, Universidade Santo Amaro (UNISA), São Paulo (SP), Brazil.

**Keywords:** Severe acute respiratory syndrome coronavirus 2 [supplementary concept], Arbovirus infections, Coinfection, Syndemic, Prognosis, COVID-19, Severity, Burden, Response, Testing, Dengue fever

## Abstract

**BACKGROUND:**

The numbers of cases of arboviral diseases have increased in tropical and subtropical regions while the coronavirus disease (COVID-19) pandemic overwhelms healthcare systems worldwide. The clinical manifestations of arboviral diseases, especially dengue fever, can be very similar to COVID-19, and misdiagnoses are still a reality. In the meantime, outcomes for patients and healthcare systems in situations of possible syndemic have not yet been clarified.

**OBJECTIVE:**

We set out to conduct a systematic review to understand and summarize the evidence relating to clinical manifestations, disease severity and prognoses among patients coinfected with severe acute respiratory syndrome coronavirus 2 (SARS-CoV-2) and arboviruses.

**METHODS:**

We conducted a rapid systematic review with meta-analysis, on prospective and retrospective cohorts, case-control studies and case series of patients with confirmed diagnoses of SARS-CoV-2 and arboviral infection. We followed the Cochrane Handbook recommendations. We searched EMBASE, MEDLINE, Cochrane Library, LILACS, Scopus and Web of Science to identify published, ongoing and unpublished studies. We planned to extract data and assess the risk of bias and the certainty of evidence of the studies included, using the Quality in Prognosis Studies tool and the Grading of Recommendations Assessment.

**RESULTS:**

We were able to retrieve 2,407 citations using the search strategy, but none of the studies fulfilled the inclusion criteria.

**CONCLUSION:**

The clinical presentations, disease severity and prognoses of patients coinfected with SARS-CoV-2 and arboviruses remain unclear. Further prospective studies are necessary in order to provide useful information for clinical decision-making processes.

**Protocol registration number in the PROSPERO database:**

CRD42020183460

## INTRODUCTION

Since the novel severe acute respiratory syndrome coronavirus 2 (SARS-CoV-2) emerged, the coronavirus disease (COVID-19) has spread worldwide. On March 11, 2020, the World Health Organization declared the outbreak of the COVID-19 disease to be a pandemic event and a Public Health Emergency of International Concern. In the meantime, its epidemiological picture has been constantly changing. Up to July 9, 2020, almost 12 million cases had been confirmed, with 545,481 deaths, in 213 countries and territories around the world, as reported to the World Health Organization (WHO).^1^^,^^2^

Amidst this pandemic, the world still needs to deal with the burden of various other diseases that present overlapping occurrences. Whether these are communicable or non-communicable, much remains to be learned regarding how to manage them all, so as to simultaneously mitigate issues relating to healthcare system saturation. In particular, countries located in tropical and subtropical regions, where arboviral diseases occur abundantly, are still dealing with these old endemics, which for some countries are epidemic diseases.^3^^−^^6^ Individuals affected by these various diseases may present clinical features that range from subclinical to severe forms, such as encephalitic or hemorrhagic forms, with very significant fatality rates.^5^ It has been estimated that more than two billion people live in environments suitable for arbovirus dissemination.^7^

Throughout the world, epidemiologists have been warning of temporal coincidence between endemic peaks and outbreaks relating to arboviruses and COVID-19.^8^^,^^9^ The constantly evolving knowledge of COVID-19 and its characteristics suggests that it and arboviral diseases share similarities with regard to clinical manifestations and laboratory findings.^4^^,^^7^ So far, dengue fever is the arboviral disease that has been found to share the largest number of clinical features with COVID-19, including the excessive systemic inflammatory response that is induced by both diseases.^4^ The effects of these diseases when a patient is infected with only one of them is already known, albeit more so with regard to arboviral diseases than to COVID-19. However, there still is a lack of information on the impact of coinfection with these diseases on patients’ clinical manifestations, the potential for severe disease and the prognosis. This knowledge is of vital importance for enabling adequate medical approaches towards these types of cases and, consequently, for applying the most appropriate treatment.

## OBJECTIVE

The aim of this rapid systematic review was to summarize the evidence that exists concerning the impact of coinfection relating to SARS-CoV-2 and arboviruses, with regard to clinical features, disease severity and prognoses among coinfected patients.

## METHODS

### Protocol and registration

The protocol for this rapid systematic review was registered within the PROSPERO (International Prospective Register of Systematic Reviews) platform, under the protocol number CRD42020183460. Additionally, we developed and published a protocol on the SciELO preprints platform (https://preprints.scielo.org/index.php/scielo/preprint/view/346).

This study was developed at the Cochrane Brazil Center and it followed the Cochrane methodology.^10^

### Eligibility criteria

#### Types of studies

Cohort studies, case-control studies and case series that described the clinical presentation, severity or prognosis of patients coinfected with SARS-CoV-2 and arboviruses were deemed to be eligible for inclusion.

#### Types of participants

Patients of any age who tested positive for SARS-CoV-2 infection and positive for any type of arboviral infection were included.

#### Types of comparators

Patients mono-infected with SARS-CoV-2 were used as comparators.

#### Outcome measurements

The primary outcomes evaluated were mortality rate, length of hospital stay and disease severity.

The secondary outcomes evaluated were clinical characteristics, length of intensive care unit stay, need for invasive mechanical ventilation, hospitalization rate and time taken to achieve clinical improvement.

### Information sources and search strategy

We developed a search strategy (Appendix 1) to retrieve eligible studies from the following databases: MEDLINE, EMBASE, the Cochrane Central Register of Controlled Trials, BVS Portal, Scopus, Web Of Science, SciELO and LILACS (Literatura Latino-Americana e do Caribe em Ciências da Saúde). Additional COVID-19 specific databases such as Epistemonikos COVID-19 L·OVE platform, ClinicalTrial.gov and the World Health Organization International Clinical Trials Registry Platform (WHO ICTRP) were also searched for ongoing studies.

To improve the range of studies that we identified, we applied specific search strategies within large open-source databases, such as Mendeley Data and Figshare. Lastly, we applied the snowballing technique, in which the reference lists of the studies selected were also screened to identify possible published papers for inclusion in this review. There were no restrictions relating to languages or publication site. All studies published before May 18, 2020 were considered within this search strategy.

### Study selection and data extraction

The titles and abstracts of citations identified through the search strategy described above were screened for eligibility by one author of this review. When duplicated citations were found, only one of them was considered for inclusion. If reports using the same participants but with different outcome measurements or different assessment time points were found, these reports would be considered as parts of only one study. Studies that clearly did not fulfill the eligibility criteria would be excluded and the remaining articles would be fully read and assessed by two authors for inclusion in the review. Disagreements between the authors, relating to this matter, would be resolved by a third author. To optimize the screening process and selection of studies, the Rayyan QCRI^11^ software was used.

We planned that two authors of this review would independently conduct the data extraction from the studies included. After that, they would together discuss any conflicts found among their results or discrepancies within this process. If necessary, a third author would mediate and resolve any conflicts. The data would be extracted through a Microsoft Excel file and would comprise information relating to study design and setting, demographic and clinical characteristics, time points used for the assessments, epidemiological characteristics, outcomes, numbers of participants, means, standard deviations, standard errors, medians, interquartile ranges, minimums, maximums, 95% confidence intervals (CI) (for continuous outcomes) and p-values, among other information.

### Risk of bias in individual studies and risk of bias across studies

We planned to perform critical appraisals on the studies included, using the Quality in Prognosis Studies (QUIPS) tool,^12^ and to assess the certainty of evidence using the GRADE approach (Grading of Recommendations Assessment, Development and Evaluation).^8^^,^^13^^,^^14^

### Summary measurements and synthesis of results

We planned to assess the possibility of pooling the results from the studies included into meta-analyses when at least two studies were sufficiently homogeneous in terms of design, participants and outcome measurements. If insufficient information or heterogeneous studies were found, we planned to summarize the results through a qualitative synthesis.

If the response of interest was provided by a continuous variable, we planned to perform the analysis in terms of the mean difference (MD) or the standardized mean difference (SMD; via Hedge's g and Cohen's d). Hazard ratios (unadjusted crude or adjusted) or odds ratios (OR) were to be pooled in cases of a dichotomous response, for hospital admission, intensive care unit admission and/or respiratory support and mortality. All the other parameters, such as standard deviations (for MD or SMD), numbers of events, relative risks or odds ratios, were planned to be pooled. In all cases, we planned to use the generic inverse variance method with a random-effects model.

### Dealing with missing data

For studies that did not provide the mean and the associated standard deviation (SD) parameters, we planned to use the information and results reported in the text or tables and to provide an inference from those findings. Additionally, we planned to contact the principal investigators of the studies included, to ask for additional data or to clarify specific concerns relating to the studies. In the absence of any response from those authors, we planned to present the data in a descriptive manner, so as to avoid making undue inferences.

### Assessment of heterogeneity

We planned to use Cochran's Q test to assess the presence of heterogeneity. We took P-values < 0.1 to be the threshold for indicating that heterogeneity was present. In addition, we planned to assess statistical heterogeneity by examining the Higgins I^2^ statistic, following these thresholds: < 25%, no heterogeneity; 25% to 49%, low heterogeneity; 50% to 74%, moderate heterogeneity; and ≥ 75%, high heterogeneity.

## RESULTS

**Figure 1:**
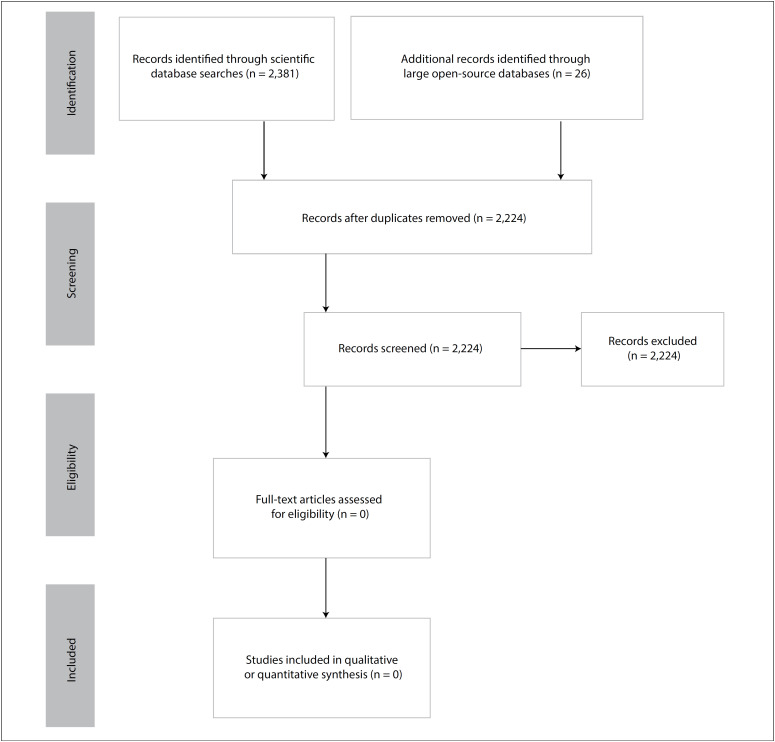
Flow diagram of the study selection process, conducted on June 20, 2020.

The search strategy developed retrieved 2,407 records (Figure 1). After removal of duplicates and screening of the citations, we were not able to find a single study that fulfilled the eligibility criteria of this systematic review.

## DISCUSSION

This rapid systematic review was the first of its kind, i.e. with the aim of summarizing the evidence relating to clinical features, disease severity and prognoses among patients coinfected with SARS-CoV-2 and arboviruses. While extraordinary attention has been given to finding effective interventions for treating patients with COVID-19, this review highlights that no significant efforts have been made to look at situations of coinfection with SARS-CoV-2 and the arboviral diseases that are already endemic in tropical and subtropical regions, and present in some temperate regions.^6^

Among over 2,000 records screened through the perspective of our search strategy, there were no studies of either observational or experimental design that had been fully performed to address any of the important aspects of coinfection between SARS-CoV-2 and arboviruses. Thus, our findings revealed an absence of published papers or other research that addressed this subject.

The limitations of this review with regard to finding eligible studies could have various explanations. Major gaps in the response to COVID-19 characterized the beginning of the pandemic.^15^ It is very likely that any initial COVID-19 patients who may have actually been coinfected were treated as presenting the COVID-19 disease only. Once a test result positive for SARS-CoV-2 had been obtained, the diagnosis would have been established and other infections may not have been considered. The opposite could also be true: if patients presented test results positive for an arboviral disease and did not progress to worsening of their health condition or symptoms, COVID-19 might not have been considered.

Part of the problem is a lack of adequate testing, for both conditions. In Brazil, for example, it has been estimated that only 23% of dengue fever cases are tested on a daily basis.^16^ However, this reality is not exclusive to the Brazilian context; the majority of the diagnoses of arboviral diseases in endemic regions, which are distinguished mostly as low-income countries, are defined through clinical-epidemiological assessment, due to lack of resources relating to the availability of testing.^17^^−^^19^

It is possible that the natural learning curve generated through responding to and managing COVID-19, including adjustment of healthcare services to the new routine, will lead to production of more reports relating to occurrences of arboviral diseases diagnosed simultaneously with COVID-19. Given that the response to the COVID-19 pandemic is still evolving, the gaps in knowledge still to be filled need to include understanding the development of coinfections between SARS-CoV-2 and arboviruses. This is critically important for development of appropriate treatment planning, in order to avoid worsening clinical status among coinfected cases.

Because of the similarities between the clinical and laboratory features of COVID-19 and arboviral diseases, differentiating between them can be a challenge,^17^^,^^20^^,^^21^ unless specific testing can be conducted. These similarities can lead to misdiagnosis of these diseases, and thus contribute to delayed treatment, thereby increasing the chances of development of greater severity of such cases and ultimately leading to death.^20^^,^^22^^,^^23^

It is noteworthy that presence of skin rashes and exanthema has been well established as having high predictive value as signs and symptoms for COVID-19.^24^^−^^27^ Skin rashes and exanthema are also present within the development of some arboviral diseases, especially dengue fever. A study conducted in Pakistan^21^ reported a misdiagnosed COVID-19 case: after two serologically negative tests for SARS-CoV-2, antibody testing for dengue fever showed positive immunoglobulin M (IgM) titers and borderline NS1 antigen results. On the other hand, a study conducted in Thailand^22^ reported a case that was initially misdiagnosed as dengue fever due to the presence of a skin rash with petechiae, which was later correlated with the COVID-19 disease. In the same way, two cases reported from Singapore^20^ were initially misdiagnosed as dengue fever through rapid tests for dengue fever that provided false-positive results. As the health condition of these patients gradually worsened, they were tested for SARS-CoV-2 and confirmed as positive cases of COVID-19.

Unfortunately, most cases of arboviral diseases relate to individuals living in low-income countries, where access to the healthcare system is difficult and of poor quality, due to lack of resources. Even worse, this scenario is faced within situations in which the healthcare system is in a fragile state, which is the reality for the majority of tropical countries.^8^^,^^28^

Ideally, rapid, sensitive, accurate and accessible tools for diagnosing the different types of arboviral diseases and COVID-19 should be considered vital. Moreover, allocation of resources to manage and respond adequately to the pandemic should be well balanced.^29^^,^^30^

Nevertheless, knowledge of the impact of this type of coinfection on patients is still unclear at best. Much remains in the realm of the unknown. Overlapping of these diseases would affect the healthcare system, which is already overwhelmed. The expression of these diseases among patients and healthcare systems in the form of a possible syndemic^31^^,^^32^ remains unclear. Therefore, we undertook a systematic search of the literature to look for outcomes from coinfection between SARS-CoV-2 and arboviruses, including their clinical presentations, disease severity and prognoses, in order to provide support for decision-makers in future scenarios of a possible syndemic.

Thinking about this matter is of vital importance, for several reasons. One of these is that there remains a need to understand what impact these types of coinfections have on the clinical manifestations, disease severity and prognoses of coinfected patients. It has already been established that both COVID-19 and dengue fever induce cytokine storms, multi-organ failure and shock.^33^ How the immune system responds to simultaneous occurrence of these diseases is a matter that has not been clarified yet.

Given the lack of evidence found, we call on researchers to conduct studies on arboviral infections within the context of the COVID-19 pandemic. Prospective cohort studies are strongly recommended within this scenario. Our research has revealed a possibly substantial public health threat that needs to be addressed. This also highlights the importance for healthcare professionals who are on the front line of providing care for patients to consider the possibility of coinfection of SARS-CoV-2 and arboviruses, especially in tropical and subtropical regions. We hope that this review may help healthcare professionals to broaden their approach to diagnosis and treatment, and that this may stimulate more vital research, in a timely manner.

## CONCLUSION

The clinical presentation, disease severity and prognoses of patients coinfected with SARS-CoV-2 and arboviruses remain unclear. Given that no eligible studies have been found to date through this systematic review, no conclusions relating to this research question can be drawn. Since this study is an ongoing systematic review, we hope to find evidence that can fill the gap in scientific information, in our subsequent publication updates.

## APPENDIX 1 Search strategies

### COCHRANE LIBRARY

#1 (COVID 19) OR (COVID-19) OR (2019-nCoV) OR (nCoV) OR (Covid19) OR (SARS-CoV) OR (SARSCov2 or ncov*) OR (SARSCov2) OR (2019 coronavirus*) OR (2019 corona virus*) OR (Coronavirus (COVID-19)) OR (2019 novel coronavirus disease) OR (COVID-19 pandemic) OR (COVID-19 virus infection) OR (coronavirus disease-19) OR (2019 novel coronavirus infection) OR (2019-nCoV infection) OR (coronavirus disease 2019) OR (2019-nCoV disease) OR (COVID-19 virus disease) OR “severe acute respiratory syndrome coronavirus 2” [Supplementary Concept] OR (Wuhan coronavirus) OR (Wuhan seafood market pneumonia virus) OR (COVID19 virus) OR (COVID-19 virus) OR (coronavirus disease 2019 virus) OR (SARS-CoV-2) OR (SARS2) OR (2019-nCoV) OR (2019 novel coronavirus)

### EMBASE

#1 ‘covid 19’/exp OR (COVID 19) OR (COVID-19) OR (2019-nCoV) OR (nCoV) OR (Covid19) OR (SARS-CoV) OR (SARSCov2 or ncov*) OR (SARSCov2) OR (2019 coronavirus*) OR (2019 corona virus*) OR (Coronavirus (COVID-19)) OR (2019 novel coronavirus disease) OR (COVID-19 pandemic) OR (COVID-19 virus infection) OR (coronavirus disease-19) OR (2019 novel coronavirus infection) OR (2019-nCoV infection) OR (coronavirus disease 2019) OR (2019-nCoV disease) OR (COVID-19 virus disease) OR (severe acute respiratory syndrome coronavirus 2) OR (Wuhan coronavirus) OR (Wuhan seafood market pneumonia virus) OR (COVID19 virus) OR (COVID-19 virus) OR (coronavirus disease 2019 virus) OR (SARS-CoV-2) OR (SARS2) OR (2019-nCoV) OR (2019 novel coronavirus)

### WEB OF SCIENCE

#1 (COVID 19) OR (COVID-19) OR (2019-nCoV) OR (nCoV) OR (Covid19) OR (SARS-CoV) OR (SARSCov2 or ncov*) OR (SARSCov2) OR (2019 coronavirus*) OR (2019 corona virus*) OR (Coronavirus (COVID-19)) OR (2019 novel coronavirus disease) OR (COVID-19 pandemic) OR (COVID-19 virus infection) OR (coronavirus disease-19) OR (2019 novel coronavirus infection) OR (2019-nCoV infection) OR (coronavirus disease 2019) OR (2019-nCoV disease) OR (COVID-19 virus disease) OR (severe acute respiratory syndrome coronavirus 2) OR (Wuhan coronavirus) OR (Wuhan seafood market pneumonia virus) OR (COVID19 virus) OR (COVID-19 virus) OR (coronavirus disease 2019 virus) OR (SARS-CoV-2) OR (SARS2) OR (2019-nCoV) OR (2019 novel coronavirus)

### SCOPUS

#1 (COVID 19) OR (COVID-19) OR (2019-nCoV) OR (nCoV) OR (Covid19) OR (SARS-CoV) OR (SARSCov2 or ncov*) OR (SARSCov2) OR (2019 coronavirus*) OR (2019 corona virus*) OR (Coronavirus (COVID-19)) OR (2019 novel coronavirus disease) OR (COVID-19 pandemic) OR (COVID-19 virus infection) OR (coronavirus disease-19) OR (2019 novel coronavirus infection) OR (2019-nCoV infection) OR (coronavirus disease 2019) OR (2019-nCoV disease) OR (COVID-19 virus disease) OR (severe acute respiratory syndrome coronavirus 2) OR (Wuhan coronavirus) OR (Wuhan seafood market pneumonia virus) OR (COVID19 virus) OR (COVID-19 virus) OR (coronavirus disease 2019 virus) OR (SARS-CoV-2) OR (SARS2) OR (2019-nCoV) OR (2019 novel coronavirus)

### PORTAL REGIONAL BVS

MH:”Infecções por Coronavirus” OR (Infecções por Coronavirus) OR (Infecciones por Coronavirus) OR (Coronavirus Infections) OR (COVID-19) OR (COVID 19) OR (Doença pelo Novo Coronavírus (2019-nCoV)) OR (Doença por Coronavírus 2019-nCoV) OR (Doença por Novo Coronavírus (2019-nCoV)) OR (Epidemia de Pneumonia por Coronavirus de Wuhan) OR (Epidemia de Pneumonia por Coronavírus de Wuhan) OR (Epidemia de Pneumonia por Coronavírus de Wuhan de 2019-2020) OR (Epidemia de Pneumonia por Coronavírus em Wuhan) OR (Epidemia de Pneumonia por Coronavírus em Wuhan de 2019-2020) OR (Epidemia de Pneumonia por Novo Coronavírus de 2019-2020) OR (Epidemia pelo Coronavírus de Wuhan) OR (Epidemia pelo Coronavírus em Wuhan) OR (Epidemia pelo Novo Coronavírus (2019-nCoV)) OR (Epidemia pelo Novo Coronavírus 2019) OR (Epidemia por 2019-nCoV) OR (Epidemia por Coronavírus de Wuhan) OR (Epidemia por Coronavírus em Wuhan) OR (Epidemia por Novo Coronavírus (2019-nCoV)) OR (Epidemia por Novo Coronavírus 2019) OR (Febre de Pneumonia por Coronavírus de Wuhan) OR (Infecção pelo Coronavírus 2019-nCoV) OR (Infecção pelo Coronavírus de Wuhan) OR (Infecção por Coronavirus 2019-nCoV) OR (Infecção por Coronavírus 2019-nCoV) OR (Infecção por Coronavírus de Wuhan) OR (Infecções por Coronavírus) OR (Pneumonia do Mercado de Frutos do Mar de Wuhan) OR (Pneumonia no Mercado de Frutos do Mar de Wuhan) OR (Pneumonia por Coronavírus de Wuhan) OR (Pneumonia por Novo Coronavírus de 2019-2020) OR (Surto de Coronavírus de Wuhan) OR (Surto de Pneumonia da China 2019-2020) OR (Surto de Pneumonia na China 2019-2020) OR (Surto pelo Coronavírus 2019-nCoV) OR (Surto pelo Coronavírus de Wuhan) OR (Surto pelo Coronavírus de Wuhan de 2019-2020) OR (Surto pelo Novo Coronavírus (2019-nCoV)) OR (Surto pelo Novo Coronavírus 2019) OR (Surto por 2019-nCoV) OR (Surto por Coronavírus 2019-nCoV) OR (Surto por Coronavírus de Wuhan) OR (Surto por Coronavírus de Wuhan de 2019-2020) OR (Surto por Novo Coronavírus (2019-nCoV)) OR (Surto por Novo Coronavírus 2019) OR (Síndrome Respiratória do Oriente Médio) OR (Síndrome Respiratória do Oriente Médio (MERS)) OR (Síndrome Respiratória do Oriente Médio (MERS-CoV)) OR (Síndrome Respiratória do Oriente Médio por Coronavírus) OR MH:C01.925.782.600.550.200$
